# Ethnopharmacological insights into *Symphytum officinale* L.: traditional uses, phytochemical composition, therapeutic potential, and clinical-toxicological implications

**DOI:** 10.3389/fphar.2026.1793084

**Published:** 2026-04-01

**Authors:** Xiaoyi Liu, Xiaochuan Huang, Xiaobin Song

**Affiliations:** College of Traditional Chinese Medicine, Shandong University of Traditional Chinese Medicine, Jinan, China

**Keywords:** chemical constituents, pharmacological properties, Symphytum officinale, toxicological profile, traditional uses

## Abstract

Symphytum officinale: L. (*S. officinale*), commonly known as comfrey, has been used in traditional medicine for over 2,000 years to treat wounds, fractures, and inflammatory conditions. This review is the first comprehensive ethnopharmacological synthesis that systematically integrates cross-cultural traditional knowledge with the latest evidence on its phytochemical profile, pharmacological mechanisms, clinical efficacy, and toxicological risks. Unlike previous fragmented reviews that addressed only isolated aspects, we followed PRISMA guidelines to analyze selected studies, with a strong emphasis on developing safe, pyrrolizidine alkaloid (PA)-depleted topical formulations that translate the plant’s classic “knitbone” reputation into modern evidence-based phytotherapy. Key bioactive constituents—allantoin, rosmarinic acid, polysaccharides, and lignans—exert anti-inflammatory, tissue-regenerative, and bone-repair effects primarily by inhibiting NF-κB and MAPK pathways and suppressing pro-inflammatory cytokines. Randomized controlled trials demonstrate that topical *S. officinale* preparations significantly outperform placebo in acute back pain, knee osteoarthritis, ankle sprains, and myalgia, while showing non-inferiority to diclofenac and an excellent safety profile. However, the presence of hepatotoxic PAs (intermedine and lycopsamine) strictly limits internal use. Topical application remains safe owing to minimal systemic absorption. By bridging historical wisdom with rigorous contemporary data and spotlighting PA-depletion strategies, this review offers a balanced framework for safe clinical application and future formulation optimization.

## 1 Introduction

**FIGURE 1:**
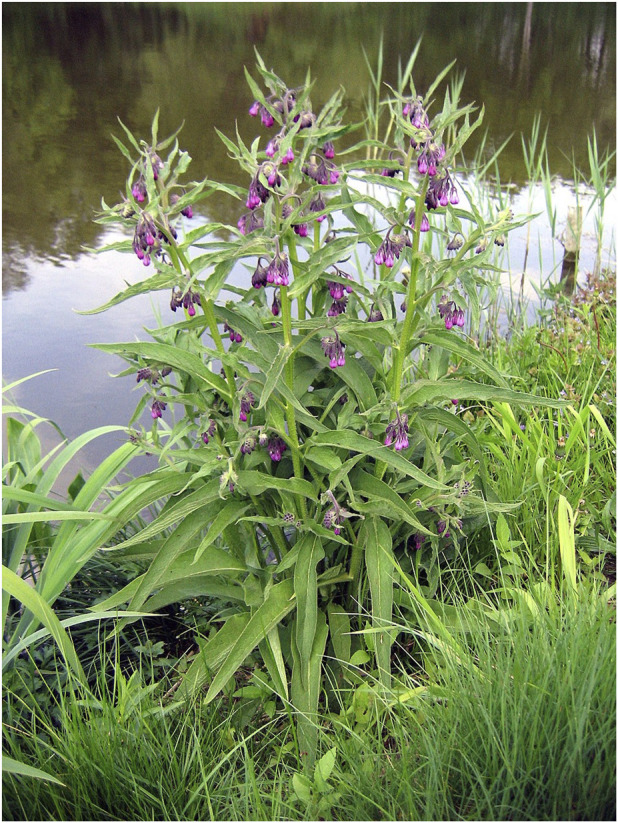
Flowering *Symphytum officinale* L. in its natural habitat. (Note: Common comfrey (*Symphytum officinale*), flowering, growing on the bank of the larger pond in the Sołtysowicki Forest, Wrocław, Poland. Photograph by Agnieszka Kwiecień, taken on 13 May 2006. Reproduced under the Creative Commons Attribution-ShareAlike 3.0 Unported license from Wikimedia Commons (https://commons.wikimedia.org/wiki/File:Symphytum_officinale_01.jpg). Permission for republication confirmed via open licensing terms).

Native to damp grasslands of Europe and parts of Asia, *S. officinale*, commonly known as comfrey, is a perennial herbaceous plant belonging to the family Boraginaceae (Fu et al., 2025; Kobrlová et al., 2022) (Figure 1). For more than two millennia, and documented since Dioscorides (ca. 50–70 AD), it has been a cornerstone of traditional herbal medicine, with its usage documented as early as ancient Greek and Roman civilizations (Al-Nimer and Wahbee, 2017; Kloter et al., 2023). Early herbalists recorded its application in treating wounds, fractures, and inflammatory disorders, attributing its effectiveness to a tissue-“knitting” capacity (Lans et al., 2007). Across European folk medicine, *S. officinale* roots and leaves were formulated into poultices, ointments, or infusions for external use on bruises, sprains, arthritis, and skin ulcers, while internal preparations alleviated gastrointestinal complaints and respiratory conditions like bronchitis (Mârza et al., 2024; Vithayathil and Edwards, 2016). Similar ethnopharmacological practices have been observed in Western Asia and parts of North America, where indigenous communities utilized *S. officinale* for musculoskeletal issues and wound healing, valuing its demulcent and regenerative properties (Antonio Pereira et al., 2023; Oster et al., 2021).

Modern scientific interest in *S. officinale* has grown substantially, driven by its diverse phytochemical composition that includes a suite of bioactive compounds such as allantoin, phenolic acids, mucilage polysaccharides and triterpenes (Kılınc et al., 2023; Shang et al., 2025; Yunusova et al., 2017). These components underpin its reported pharmacological activities, including anti-inflammatory, skin-protective, and antioxidant effects, which have been validated by *in vitro* and *in vivo* studies (Kloter et al., 2023; Mahmoudzadeh et al., 2022; Zhao et al., 2024). Clinical research has further confirmed its efficacy in topical applications, demonstrating benefits for acute back pain, osteoarthritis, and wound management, with formulations typically designed to mitigate potential risks (Khan et al., 2020; Oltean et al., 2014; Staiger, 2012). However, the presence of pyrrolizidine alkaloids (PAs), most notably lycopsamine and intermedine, presents substantial toxicological risks (Wang et al., 2022; Zakaria et al., 2021). Upon bioactivation, these compounds induce hepatotoxicity, leading to regulatory restrictions on internal use and emphasizing the need for PA-reduced products (Neuman et al., 2015). As a technologically relevant medicinal plant, S. officinale extracts are increasingly applied in cosmetic formulations, veterinary wound care, and sustainable agriculture as a protein-rich feed supplement, underscoring its economic and ecological importance beyond classical phytotherapy (Oster et al., 2021; ESCOP, 2014).

Previous reviews have addressed isolated aspects of *S. officinale*, such as traditional uses, phytochemistry, clinical applications, or toxicity. However, earlier comprehensive monographs provided foundational but fragmented assessments (German Commission E, 1990; British Herbal Pharmacopoeia, 1996; ESCOP, 2009; ESCOP, 2014). This review fills these gaps by providing a balanced, updated framework that links historical practices to modern evidence-based phytotherapy, with particular emphasis on safe topical applications and future formulation optimization. This review first synthesizes the multifaceted nature of *S. officinale*, covering its traditional ethnomedicinal uses, detailed phytochemical makeup, pharmacological mechanisms, clinical evidence, and toxicological profile. By integrating these elements, this review aims to provide a balanced perspective on the plant’s therapeutic potential while highlighting critical safety considerations, especially in the context of ethnopharmacology where traditional knowledge intersects with contemporary scientific validation. Such an integrated approach is essential for guiding the safe utilization of *S. officinale* and inspiring further research into optimized formulations that harness its benefits without compromising health.

### 1.1 Methodology

**FIGURE 2:**
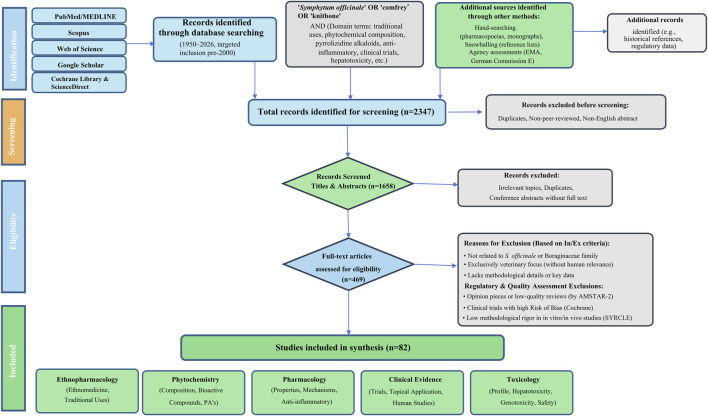
PRISMA flow diagram of the systematic literature search and selection process.

To systematically identify and synthesize relevant literature on the ethnopharmacological, phytochemical, pharmacological, clinical, and toxicological aspects of *Symphytum officinale* L., a comprehensive search was conducted across PubMed/MEDLINE, Scopus, Web of Science, Google Scholar, Cochrane Library, and ScienceDirect. The PRISMA-compliant selection process is shown in Figure 2. In total, 2,347 records were retrieved. After removal of 689 duplicates, 1,658 titles and abstracts were screened, of which 1,189 were excluded as clearly irrelevant. Full-text assessment was performed on 469 articles; 387 were subsequently excluded for not meeting inclusion criteria (non-peer-reviewed, insufficient methodological detail, purely veterinary focus, or lacking human-health relevance). Ultimately, 82 studies were included for synthesis, distributed across domains as follows (with some overlap): ethnopharmacology/traditional uses (12), phytochemistry (33), pharmacological properties (30), clinical applications (12), and toxicological profile (10). The primary publication filter was peer-reviewed articles from 2000 to 2026, with targeted inclusion of pre-2000 foundational references., monographs (German Commission E, ESCOP), and (European Medicines Agency, 2011; European Medicines Agency, 2015) through hand-searching and snowballing from reference lists.

Key search terms were structured using Boolean operators (AND/OR/NOT) and included combinations such as “*Symphytum officinale*” OR “comfrey” OR “knitbone,” paired with domain-specific qualifiers: “traditional uses” OR “ethnomedicine” OR “folk medicine” OR “ethnopharmacology” for historical applications; “phytochemical composition” OR “bioactive compounds” OR “phenolics” OR “lignans” OR “pyrrolizidine alkaloids” OR “polysaccharides” for chemistry; “pharmacological properties” OR “anti-inflammatory” OR “bone repair” OR “skin protection” OR “anti-cancer” OR “antioxidant” for mechanisms; “clinical trials” OR “randomized controlled trials” OR “topical application” OR “musculoskeletal disorders” OR “osteoarthritis” OR “back pain” OR “ankle sprains” for human studies; and “toxicological profile” OR “hepatotoxicity” OR “pyrrolizidine alkaloids” OR “genotoxicity” OR “carcinogenicity” OR “adverse effects” for safety concerns. To expand historical coverage as recommended, we explicitly incorporated key pre-2000 monographs and reviews: German Commission E (Kommission, 1990; Blumenthal, 1998), British Herbal Pharmacopoeia (1996), ESCOP monographs (2009 edition), and EMA assessment reports (2011 draft).

**Inclusion criteria**: (1) peer-reviewed original articles, systematic reviews, meta-analyses, clinical trials, *in vitro*/*in vivo* studies, ethnobotanical surveys, or case reports directly addressing *S. officinale* or closely related *Symphytum* species; (2) focus on ethnopharmacological, phytochemical, pharmacological, clinical, or toxicological aspects relevant to human health; (3) published 2000–2026; (4) English full text or English abstract. **Exclusion criteria:** (1) non-peer-reviewed sources, conference abstracts without full text, duplicates, or opinion pieces; (2) studies unrelated to the Boraginaceae family or exclusively veterinary without human relevance; (3) articles lacking methodological details or full-text access. Full-text review, supplemented by snowballing from reference lists of seminal works, resulted in over 120 sources for synthesis. Quality assessment employed tools such as the Cochrane Risk of Bias for clinical trials, SYRCLE for animal studies, and AMSTAR-2 for reviews; regulatory documents (EMA, German Commission E, European Pharmacopoeia) were evaluated for authoritative status. Although the review was not prospectively registered in PROSPERO, the methodology fully adheres to PRISMA standards. This expanded temporal scope ensures capture of foundational toxicity and clinical evidence while enabling critical evaluation of evolving regulatory perspectives *in vitro* and *in vivo* (Figure 2).

## 2 Traditional uses

*S. officinale* boasts a rich, cross-continental legacy in traditional medicine spanning millennia, rooted in its observed ability to promote tissue repair and alleviate discomfort from various ailments (Antonio Pereira et al., 2023). Historical records trace its use back to ancient civilizations, where herbalists valued it primarily for addressing wounds, fractures, and inflammatory conditions (Staiger, 2013). This early focus on its astringent and regenerative properties laid the groundwork for its enduring role in folk remedies, which evolved through the Middle Ages. During this period, *S. officinale* was commonly applied to burns, contusions, and internal disorders such as tonsillitis, metrorrhagia, phlebitis, and gastroduodenal complaints (Salehi et al., 2019). While these applications were empirically derived, they reflect a reliance on *S. officinale*’s mucilaginous and anti-inflammatory traits. Modern phytochemical studies have retrospectively associated these empirical effects with compounds such as allantoin and rosmarinic acid; however, traditional practitioners relied solely on empirical observations and experiential knowledge rather than identified chemical constituents (Salehi et al., 2019).

From antiquity to regional folk practices, *S. officinale*’s traditional uses expanded across Europe, becoming a staple in herbal traditions for musculoskeletal and dermatological issues. Ethnopharmacological surveys covered Germany, Lithuania, Poland, and the United Kingdom. They reveal frequent use of the plant for sprains, strains, rheumatic pain, and joint swelling. External preparations were the primary delivery route, as they capitalized on the plant’s soothing properties (Frost, O'Meara and MacPherson, 2014). In rural Lithuania, for example, *S. officinale* roots were prepared as teas or ointments to relieve bone pain and fractures, while in Navarra, Spain, both *S. officinale* and related species like *S. tuberosum* were incorporated into clay balms for rheumatism and sprains (Salehi et al., 2019). Similarly, Italian folk medicine documented its decoctions for antidiarrheal purposes. In Switzerland, *S. officinale* featured in veterinary remedies for animal wounds, which reflects a broader veterinary role that parallels its use in humans (Trifan et al., 2024). These practices extended to Eastern Europe and Asia, including the Caucasus (where species like *S. caucasicum* were used for inflammatory disorders), Ukraine (especially the Carpathians, for bone pain and fractures), and East and Central Asia (for antioxidant and anti-complement activities in treating wounds and inflammation) (Mahmoudzadeh et al., 2022; Trifan et al., 2024; Salehi et al., 2019). Further afield, ethnographic studies in Mexico note the use of infused aerial parts for hepatic disturbances and rheumatism, while in Brazil, infusions targeted gastritis and ulcers. In the United States, including among Native American communities, *S. officinale* was valued for skin problems and as a tonic (Antonio Pereira et al., 2023). This geographic diversity underscores *S. officinale*’s adaptability, yet it also reveals cultural nuances, such as preferences for roots over leaves in certain regions, shaped by local biodiversity and knowledge transmission (Salehi et al., 2019).

**FIGURE 3:**
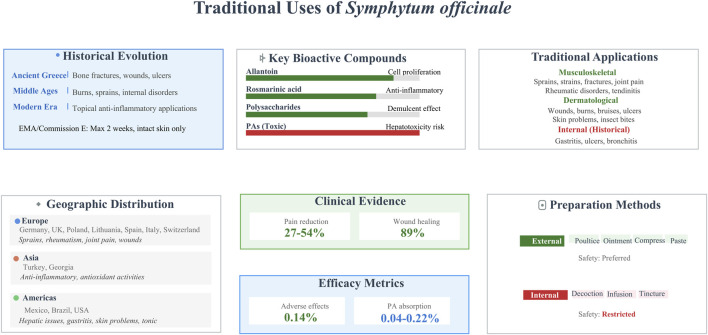
A comprehensive infographic of traditional uses, clinical efficacy, and safety risks of *S. officinale*. (Note: This infographic provides a structured summary of *S. officinale*, covering its historical medicinal evolution, key bioactive compounds (including toxic pyrrolizidine alkaloids), global distribution, preparations, and safety considerations (with restricted internal use). The 27%–54% figures originate from clinical studies on back pain reduction (e.g., Pabst et al., 2013), and the 89% from wound size reduction (e.g., Štepán et al., 2014). Quantitative metrics for adverse events (0.14%) and alkaloid absorption (0.04%–0.22%) are included to contextualize benefit-risk balance).

Traditional preparations of *S. officinale* varied widely, reflecting both ingenuity and the plant’s versatile phytochemistry, though a consistent shift toward prioritizing topical over internal use emerged in later periods, driven by growing safety concerns (Inkeniene and Vaiciuleviciene, 2023). Fresh or dried roots, leaves, or aerial parts were often pulverized into mashes, poultices, compresses, pastes, or collars for direct application to affected areas—aimed at reducing swelling and promoting healing in conditions like bruises, tendinitis, knee injuries, insect bites, mastitis, and non-active gonarthrosis (Frost et al., 2014). In some cases, ointments containing liquid extracts (typically with ethanol in varying concentrations) were formulated to enhance skin penetration. European pharmacopeias, for instance, standardized *S. officinale* root extract ointments for treating blunt traumas and degenerative rheumatic disorders (Trifan et al., 2024). Historically, internal use was more prevalent, with *S. officinale* integrated into salads, soups, juices, milk, bread, or prepared as decoctions, infusions, tinctures, and potions to address pulmonary issues, bronchitis, and gastrointestinal complaints (Antonio Pereira et al., 2023; Lans et al., 2007). However, contemporary ethnopharmacological assessments have curtailed such internal applications. These assessments draw on regulatory evaluations from bodies such as the European Medicines Agency and the German Commission E (Trifan et al., 2024; Staiger, 2013). These authorities recognize *S. officinale*’s well-established external use but recommend limiting topical use to short durations (typically up to 10 days or 4–6 weeks per year) on intact skin to minimize risks. Ethnopharmacological studies further highlight broader implications of *S. officinale* use. These include its role in sustainable agriculture and animal nutrition as a feed supplement. Rigorous safety protocols remain essential for such applications. *In vitro* Global ethnobotanical surveys confirm its occasional use as a feed supplement in sustainable agriculture and animal nutrition (Oster et al., 2021). Rigorous safety protocols remain essential for such applications, and integration into local healing systems for osteoarthritis and wounds has been documented in various regions, mirroring European practices (Salehi et al., 2019). Overall, while traditional uses confirm *S. officinale*’s utility in addressing inflammatory conditions and promoting tissue regeneration, ongoing analyses advocate for refined extraction methods to mitigate risks. This ensures *S. officinale* retains relevance in phytotherapy without compromising user safety (Figure 3) (Table 1).

**TABLE 1:** Traditional uses of the *S. officinale* worldwide.

Country/Region	Plant part used	Preparation method	Traditional uses	References
Germany	Roots, Leaves	Ointments, Poultices	Sprains, rheumatic pain, joint swelling	Frost et al. (2014), Salehi et al. (2019)
Lithuania	Roots	Teas, Ointments	Bone pain, fractures, contusions	Salehi et al. (2019); Trifan et al. (2024)
Poland	Aerial parts	Decoctions	Rheumatic disorders, wounds	Frost et al. (2014); Trifan et al. (2024)
United Kingdom	Leaves	Compresses	Sprains, bruises, skin issues	Frost et al. (2014); Salehi et al. (2019)
Spain (Navarra)	Roots, Aerial parts	Clay balms	Rheumatism, sprains	Salehi et al. (2019); Trifan et al. (2024)
Italy	Whole plant	Decoctions	Antidiarrheal, gastrointestinal complaints	Trifan et al. (2024); Salehi et al. (2019)
Switzerland	Roots	Veterinary ointments	Animal wounds, inflammation	Trifan et al. (2024); Lans et al. (2007)
Ukraine	Roots	Teas, Compresses	Bone fractures, joint pain	Trifan et al. (2024); Salehi et al. (2019)
East/Central Asia	Aerial parts, Roots	Infusions, Extracts	Antioxidant support, anti-inflammatory for wounds	Mahmoudzadeh et al. (2022); Trifan et al. (2024)
Mexico	Aerial parts	Infusions	Hepatic disturbances, rheumatism	Salehi et al. (2019); Trifan et al. (2024)
Brazil	Aerial parts	Infusions	Gastritis, ulcers	Salehi et al. (2019); Trifan et al. (2024)
United States	Leaves, Roots	Tonics, Poultices	Skin problems, general tonic	Salehi et al. (2019); Trifan et al. (2024)
Jamaica	Roots	Tonics	Osteoarticular pain	Salehi et al. (2019)

Critically, while ethnobotanical surveys and historical texts provide a rich descriptive panorama of S. officinale’s cross-cultural applications, the evidence remains largely anecdotal and qualitative. Most surveys suffer from recall bias, lack of standardization in preparation methods, and absence of dose–response data. Additionally, pre-2000 monographs (German Commission E, 1990; British Herbal Pharmacopoeia, 1996) robustly support external use for minor sprains and bruises on the basis of long-standing traditional use, yet explicitly prohibit internal consumption due to pyrrolizidine alkaloid (PA) risks. The evidence base shows consistent topical indications across continents but fails to provide high-quality quantitative validation for internal uses; older studies lack dose-response data and standardization.

## 3 Phytochemistry

### 3.1 Extraction and isolation techniques

Various technological approaches have been employed to obtain extracts from *S. officinale*, focusing on roots, leaves, and aerial parts. Common methods include solvent extraction using ethanol, methanol, or water, often assisted by ultrasound, microwaves, or hot-water techniques for improved yield and selectivity (Le et al., 2021; Lou et al., 2023; Shang et al., 2018). For instance, ultrasonic enzyme-assisted extraction has been used to isolate polysaccharides, while natural deep eutectic solvents (NADES) like betaine-urea selectively extract phenolics while minimizing pyrrolizidine alkaloid content (Syarifah et al., 2023). Advanced fractionation techniques, such as liquid chromatography coupled with high-resolution tandem mass spectrometry (LC-ESI-FT-MS^n^) and nuclear magnetic resonance spectroscopy, facilitate the isolation and structural elucidation of compounds (D'Urso et al., 2020; Trifan et al., 2021b). Ion-exchange chromatography (e.g., DEAE-cellulose) and gel filtration are applied for polysaccharide purification, ensuring low protein contamination and high molecular weight fractions (Chen et al., 2018; Duan et al., 2018). These methods not only enhance extraction efficiency but also support the development of PA-depleted products for safer applications. These investigations highlight the plant’s rich phytochemical composition, encompassing phenolic derivatives, lignans, PAs, and miscellaneous constituents such as purine derivatives and terpenoids.

### 3.2 Hydroxycinnamic acids

Hydroxycinnamic acids represent one of the most abundant classes in *S. officinale*, particularly in root and callus culture extracts, where they play a pivotal role in mediating the plant’s antioxidant and anti-inflammatory activities. Rosmarinic acid, a caffeic acid depside, stands out as a major marker compound. Its concentration in ethanolic root extracts can reach up to 45.70 μg/mL (Le et al., 2021). Monomeric hydroxycinnamic acids including caffeic acid, chlorogenic acid, p-coumaric acid, and p-hydroxybenzoic acid have been isolated, mainly in root extracts. Caffeic acid ethyl ester was identified for the first time in *S. officinale* roots (D’Urso et al., 2020). Another novel hydroxycinnamic acid from root callus cultures, m-methoxybenzoic acid, reaches levels of 30.05 μg/mL (Le et al., 2021). Notably, this class of compounds exhibits structural diversity, including oligomers such as danshensu and salvianolic acid derivatives. Extraction solvents, such as natural deep eutectic solvents (NADES), can selectively boost phenolic yields while minimizing toxic co-extracts (Syarifah et al., 2023).

### 3.3 Lignans

Lignans, especially arylnaphthalene-type lignans, add complexity to *S. officinale*’s phytochemical profile, with most studies focusing on roots as the source organ. Comfreyn A—a rare arylnaphthalene lignan bearing a δ-lactone ring—was recently isolated from root extracts via LC-ESI-FT-MS^n^-guided fractionation, marking its first identification in the Boraginaceae family (D’Urso et al., 2020). Similarly, globoidnan A, globoidnan B, and rabdosiin serve as novel phenolic markers in European-sourced *S. officinale* roots. Their quantitative levels are influenced by post-harvest storage, peaking at 1–3 months before declining (Trifan et al., 2021c). Additional lignans, including ternifoliuslignan D and 3-carboxy-6,7-dihydroxy-1-(3′,4′-dihydroxyphenyl)-naphthalene, have been newly reported in *S. officinale* roots (D’Urso et al., 2020; Le et al., 2021). Malaxinic acid, a phenolic acid derivative, is also present (D’Urso et al., 2020). Storage time affects their stability, which is a critical consideration for quality control in pharmaceutical formulations.

### 3.4 Alkaloids

PAs represent a double-edged sword in *S. officinale*’s phytochemical profile, predominantly studied in roots due to their higher concentration in this organ compared to aerial parts or leaves. Major isolated PAs include intermedine, lycopsamine, acetylintermedine, acetyllycopsamine, symphytine, and their N-oxides. Quantified in roots from various European regions, their levels fluctuate with storage duration (Trifan et al., 2021a). In *S. officinale roots*, the primary PAs are lycopsamine (up to 0.5–1.5 mg/g dry weight) and intermedine (0.3–1.0 mg/g), often accompanied by their acetylated forms and symphytine (0.1–0.5 mg/g). N-oxides, such as symphytine-N-oxide, contribute to total PA content (up to 2–5 mg/g) (Trifan et al., 2021c; Luca et al., 2024). These 1,2-unsaturated necine-type PAs are predominantly found in the roots, with concentrations varying by geographical origin, harvest time, and post-harvest processing.

### 3.5 Flavonoids and triterpenoid

**FIGURE 4:**
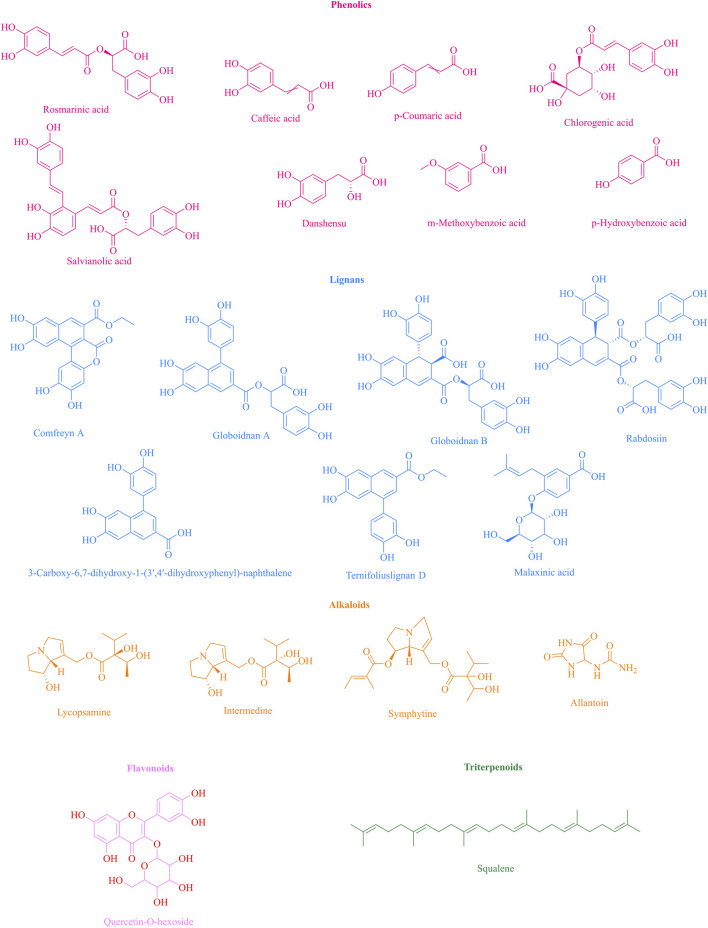
Chemical structures of major bioactive compounds isolated from *S. officinale.* (Note: This figure presents the chemical structures of key phytochemicals identified in Symphytum officinale, classified by their functional classes: phenolics, lignans, alkaloids, flavonoids, and triterpenoids. These compounds underpin the plant’s traditional medicinal activities, while the inclusion of toxic alkaloids highlights its critical safety considerations).

Flavonoids in *S. officinale*, primarily identified in root and leaf extracts, include quercetin-O-hexoside and kaempferol derivatives, detected via LC-HRMS/MS analyses (Trifan et al., 2021b; Kılınc et al., 2023). Squalene, a triterpenoid in callus cultures, is also present (Le et al., 2021). Their concentrations are generally lower than hydroxycinnamic acids (Figure 4).

### 3.6 Polysaccharides

In recent years, polysaccharides have become one of the most intensively studied macromolecular constituents of *S. officinale* aerial parts or roots. Early phytochemical investigations established that water-soluble polysaccharides can be efficiently extracted from the aerial parts or roots of *S. officinale* via hot-water extraction, followed by ethanol precipitation and deproteinization. Subsequent purification strategies, with DEAE-cellulose ion-exchange chromatography and gel filtration as the most common ones, have shown that *S. officinale* polysaccharides are heterogeneous fractions. These fractions have relatively high molecular weights and low protein contamination, consistent with the characteristics of typical plant-derived non-starch polysaccharides (Chen et al., 2018; Duan et al., 2018).

Structural characterization studies consistently demonstrate that *S. officinale* polysaccharides are primarily composed of galactose, arabinose, glucose, and significant amounts of galacturonic acid. This composition points to the presence of pectic polysaccharides, which are enriched in arabinogalactan and homogalacturonan domains. Monosaccharide analysis combined with molecular weight determination has revealed that these polymers typically exhibit broad molecular weight distributions, reflecting structural complexity and potential heterogeneity in branching patterns (Cao et al., 2021).

From a critical standpoint, existing research on *S. officinale* polysaccharides remains largely descriptive, with limited elucidation of fine structural features such as glycosidic linkages, branching patterns, and conformational properties. Furthermore, correlations between structure and bioactivity are still tentative, as most biological evaluations have been conducted using crude or partially purified fractions. Future studies integrating advanced spectroscopic techniques with well-designed bioassays will be essential to clarify structure–function relationships and identify the specific polysaccharide motifs responsible for observed activities (Table 2).

**TABLE 2:** Chemical characteristics and biological activities of polysaccharides isolated from *S. officinale*.

Name	Composition	Molecular weight	Biological activities	Ref.
*S. officinale* polysaccharides (SOP)	Galacturonic acid (17.01 mol%), arabinose (16.15 mol%), galactose (20.83 mol%), glucose (46.01 mol%)	6.25 kDa	Resistant to gastrointestinal digestion and modulates chicken cecal microbiota	Cao et al. (2021)
*S. officinale* root polysaccharides (HW-CRPs)	Galacturonic acid (3.14%), glucose (68.92%), galactose (16.36%), arabinose (11.58%)	5811.42 Da	Antioxidant activity; α-glucosidase inhibition activity	Chen et al. (2018)
*S. officinale* polysaccharides (HW-CPs)	Galacturonic acid (7.45%), glucose (67.18%), galactose (17.36%), arabinose (8.01%)	997.20 × 10^4^ Da and 2878.33 Da	Antioxidant activity; α-glucosidase inhibition activity	Duan et al. (2018)
*S. officinale* root polysaccharides (HD-CRPs)	Galacturonic acid, glucose, galactose, arabinose (molar ratio: 1:15.27:3.41:2.37)	7042.63 Da	Antioxidant activity	Shang et al. (2018)
*S. officinale* polysaccharides (CPs)	Mannose (2.25%), rhamnose (3.79%), galacturonic acid (19.50%), glucose (60.13%), galactose (6.63%), arabinose (7.70%)	3.65 × 10^4^ Da	Improves laying hen production performance; enhances nutrient digestibility; modulates cecal microbiota; increases SCFAs production	Zhou et al. (2022)

Activities confirmed for polysaccharides specifically isolated from *S. officinale*.

Phytochemical profiling of S. officinale has advanced considerably with modern analytical techniques (LC-MS, NMR), yet substantial gaps persist. While modern LC-MS/NMR has advanced profiling, older studies (pre-2000) often lacked GLP compliance and PA quantification; post-harvest storage effects are documented but rarely controlled in commercial products. The data convincingly identify allantoin, rosmarinic acid, and polysaccharides as key actives underpinning traditional claims, yet fail to establish clear structure–activity relationships for most minor constituents or to quantify batch-to-batch variability. Scientific quality of older studies is often low (no GLP compliance, inadequate validation of methods). Future research needs to prioritize PA-depleted, chemically standardized extracts and metabolomic fingerprinting across geographic origins to support regulatory acceptance and technological applications (e.g., cosmetics, nutraceuticals).

## 4 Pharmacological properties

The pharmacological properties of *S. officinale* are primarily attributed to its extracts and isolated bioactive compounds, including phenolic acids, lignans, polysaccharides, and purine derivatives like allantoin. The following sections detail the activities observed in studies using either whole plant extracts, fractions, or specific compounds isolated from *S. officinale*, as indicated. Where relevant, the source (e.g., isolated compound vs. extract) is clarified to distinguish between effects of pure substances and potential synergistic actions in complex mixtures.

### 4.1 Anti-inflammatory activity

Many of the anti-inflammatory activities reported for *S. officinale* derive from studies on root or leaf extracts, which contain mixtures of compounds, although some effects have been attributed to isolated substances such as rosmarinic acid, caffeic acid derivatives, and lignans. *In vitro* and *in vitro* studies using specific cell models have yielded substantial evidence of *S. officinale*’s anti-inflammatory capacity.

For example, a root extract suppressed interleukin-1β (IL-1β)-induced expression of adhesion molecules (E-selectin, vascular cell adhesion molecule-1 [VCAM-1], and intercellular adhesion molecule-1 [ICAM-1]) in primary human umbilical vein endothelial cells (HUVECs) and also reduced cyclooxygenase-2 (COX-2) levels. Key compounds such as caffeic acid ethyl ester and the novel lignan comfreyn A inhibited E-selectin at concentrations of 64 μM and 50 μM, respectively (D’Urso et al., 2020). Similarly, PA- and mucilage-depleted root extracts retained the ability to inhibit pro-inflammatory cytokine release (IL-1β, IL-8, and TNF-α) in LPS-stimulated neutrophils, confirming that anti-inflammatory efficacy is preserved post-toxin removal (Trifan et al., 2023). Leaf extracts further exhibited potent inhibition of inducible nitric oxide synthase (iNOS), COX-2, IL-1β, IL-6, and TNF-α in LPS-stimulated RAW264.7 macrophages, an effect primarily attributed to rosmarinic acid (Lou et al., 2023). These findings demonstrate that *S. officinale* derivatives modulate inflammatory mediators at the cellular level, likely through interference with key signaling pathways.

Building on cellular data, *in vivo* models have validated the translational potential of these anti-inflammatory effects. In a dextran sulfate sodium-induced ulcerative colitis mouse model, *S. officinale* root pectin at 100–400 mg/kg alleviated symptoms (weight loss, diarrhea, colon shortening), reduced serum TNF-α and IL-6 levels, and elevated IL-10. It also enhanced expression of tight junction protein zonula occludens-1 (ZO-1) and mucin 2 (Muc2), restored colonic epithelial integrity, and modulated gut microbiota—reducing pro-inflammatory genera such as *Oscillibacter* and *Alistipes* (Liu et al., 2024). Another study used a paste of *S. officinale* tincture and calcium hydroxide in rats with experimental jaw bone defects, observing accelerated bone remodeling and reduced inflammation *via* morphological and densitometric analyses, without hepatotoxicity or damage to surrounding tissues (Kostyiuk et al., 2021). Additionally, caffeic acid oligomers from *S. officinale* roots inhibited TNF-α, IL-8, and IL-1β release in LPS-stimulated neutrophils, highlighting the role of these polyphenols in attenuating systemic inflammation (Trifan et al., 2020). These animal studies confirm *S. officinale*’s ability to promote tissue repair and dampen inflammatory responses *in vivo*, often linked to improved barrier function and microbial balance.

Mechanistic studies reveal that *S. officinale*’s anti-inflammatory effects frequently target the nuclear factor-kappa B (NF-κB) pathway—a central regulator of inflammation. A root extract impaired NF-κB activation in HUVECs through two key mechanisms: inhibiting IκB kinase (IKK)1/2-mediated IκBα degradation and interfering with p65 subunit nucleocytoplasmic shuttling and transactivation, even in mucilage-depleted fractions (Seigner et al., 2019). This dual blockade aligns with macrophage model findings, where leaf extracts suppressed NF-κB and mitogen-activated protein kinase (MAPK) signaling, reducing cytokine production (Lou et al., 2023). Furthermore, detoxified extracts preserved anti-inflammatory potency (Syarifah et al., 2023), demonstrating that refined processing can enhance both safety and bioactive yield.

However, most *in vitro*/*in vivo* data derive from crude extracts with supra-physiological concentrations unattainable in topical use; many lack blinding or dose-response curves. PA-depleted extracts preserve activity (Trifan et al., 2023), but long-term bioavailability remains underexplored. Evidence supports NF-κB/MAPK inhibition but provides only weak support for systemic effects due to inadequate pharmacokinetic data.

### 4.2 Bone repair

*S. officinale* has long been recognized for its potential to accelerate bone healing, primarily attributed to bioactive compounds such as allantoin and rosmarinic acid—agents that promote tissue regeneration and reduce inflammation. Recent pharmacological studies have explored its efficacy in enhancing bone repair (particularly osseointegration and fracture recovery) using *in vitro* and *in vivo* models, providing empirical evidence of osteogenic properties, including improved cell proliferation, differentiation, and bone density around implants or defects.

*In vitro* experiments highlight *S. officinale*’s ability to stimulate osteogenic differentiation in mesenchymal stem cells (MSCs)—key regulators of bone formation. For instance, human bone marrow-derived MSCs treated with *S. officinale* extracts exhibited enhanced osteogenesis, as indicated by elevated expression of RUNX2, osteopontin, and osteocalcin, alongside increased alkaline phosphatase (ALP) activity over a 2 week period. The treatment induced a dose-dependent increase in mineralization, with optimal effects at concentrations that avoided cytotoxicity (Dey et al., 2020). Similarly, rat bone marrow MSCs treated with homeopathic *S. officinale* dilutions showed significantly higher ALP activity and calcium nodule formation compared to standard osteogenic media. Quantitative assays revealed ALP levels increased by 4.04-fold on day 7 and 7.86-fold on day 21, highlighting its potential as a cost-effective alternative to synthetic osteogenic inductors (Vaezi et al., 2020). These findings suggest *S. officinale* modulates signaling pathways involved in osteoblast maturation, possibly via anti-inflammatory mechanisms that create a favorable microenvironment for bone matrix deposition.

Complementing these cellular insights, surface modification studies have explored *S. officinale*’s role in enhancing implant integration. An *in vitro* assessment of titanium discs treated with *S. officinale* gel (alone or combined with photofunctionalization) demonstrated superior adhesion and proliferation of MG-63 osteoblast-like cells compared to untreated controls. ANOVA statistical analysis confirmed significantly higher cell differentiation in *S. officinale*-exposed groups, with proliferation rates markedly elevated (Khan et al., 2020). This indicates *S. officinale* extracts improve bone-implant contact by fostering osteogenic cell retention, likely stemming from its nutrient-rich profile that supports callus formation and granulation tissue development.

Animal models further validate these observations, particularly in evaluating bone density and mechanical strength around titanium implants. In rats, oral administration of homeopathic *S. officinale* (6 cH) post-implantation increased radiographic bone density (measured *via* digital subtraction radiography). At 14 days, treated groups exhibited a mean gray shade of 175.3—significantly higher than controls (146.2)—indicating enhanced early-stage bone gain (Sakakura et al., 2008). Extending this, another study reported that the same dilution increased removal torque and bone density, with torque values peaking at 56 days, suggesting sustained osteogenic stimulation (predominantly in early healing stages) (Spin-Neto et al., 2010). Additionally, a paste of *S. officinale* tincture and calcium hydroxide promoted bone regeneration in rat mandibular defects, with remodeling comparable to natural healing but with pronounced anti-inflammatory effects. Morphological evaluations showed reduced tissue inflammation and stimulated osteosynthesis without hepatotoxicity or jaw tissue damage, positioning the paste as a viable therapy for chronic apical periodontitis (Kostyiuk et al., 2021).

Collectively, these studies demonstrate *S. officinale*’s multifaceted role in bone healing. Its roles range from direct cellular osteogenic induction to supportive anti-inflammatory effects that mitigate post-injury complications. While *in vitro* and animal data are promising, clinical translation requires caution—raw *S. officinale*’s PA content necessitates purified or diluted extracts to avoid hepatotoxicity. Future research should prioritize randomized controlled trials to assess long-term efficacy and safety in fracture management or implant dentistry, potentially integrating *S. officinale* with modern biomaterials for optimized regenerative outcomes.

### 4.3 Skin protection

The skin-protective effects of *S. officinale* are largely observed in studies using extracts, with contributions from isolated compounds like rosmarinic acid, salvianolic acids, and allantoin, as well as synergistic effects in nanoparticle formulations.

A core aspect of *S. officinale*’s skin-protective capacity lies in its ability to mitigate oxidative stress and promote cellular proliferation in dermal cells. Root extracts have been shown to possess robust free radical scavenging activity, primarily attributed to phenolic compounds such as rosmarinic acid and salvianolic acids. For example, an *in vitro* study on human skin fibroblasts found the extract not only enhanced cell viability and metabolism but also exhibited high antioxidant potential in DPPH and FRAP assays, even outperforming the effects of isolated phenolic acids alone (Sowa et al., 2018). This suggests synergistic interactions among the extract’s components, where the combined presence of allantoin and phenolics amplifies cytoprotective outcomes. Such findings imply *S. officinale* extracts could serve as adjuncts in therapies targeting age-related skin degeneration or environmental stressors.

Beyond direct cellular benefits, *S. officinale* extracts have demonstrated efficacy in wound-healing models by accelerating tissue regeneration and reducing inflammation. In a comparative analysis, creams and gels formulated with aqueous *S. officinale* root extract outperformed preparations containing pure allantoin in restoring skin barrier function following artificial irritation in human volunteers. Specifically, extract-based formulations improved skin hydration, reduced erythema, and lowered transepidermal water loss more effectively. Creams showed superior performance in this regard, highlighting the role of additional constituents (polysaccharides and phenolic acids) in enhancing anti-irritant activity (Savić et al., 2015). Complementing this, animal studies using rat excisional wound models revealed that oil-in-water creams incorporating 20% *S. officinale* root extract accelerated wound closure and achieved complete epithelialization within 14 days. This efficacy correlated with high levels of allantoin, rosmarinic acid, and salvianolic acids, which conferred antimicrobial activity against pathogens such as *Staphylococcus aureus* and *Escherichia coli* (Mârza et al., 2024). These observations underscore the extract’s multifaceted role in modulating the wound microenvironment—likely by inhibiting microbial colonization while stimulating fibroblast activity.

An innovative dimension of *S. officinale*’s skin-protective profile emerges from nanoparticle formulations. Silver nanoparticles derived from *S. officinale* significantly attenuated UVB-induced photoaging in human keratinocytes by downregulating matrix metalloproteinase-1 (MMP-1) and interleukin-6 (IL-6), while upregulating procollagen type I expression (Singh et al., 2018). This mechanism likely involves the nanoparticles’ ability to scavenge reactive oxygen species (ROS) generated by UV exposure, thereby preserving extracellular matrix integrity.

Furthermore, interactions with the skin microbiota add another layer to *S. officinale*’s protective effects, as microbial metabolism can modulate the bioavailability and toxicity of its constituents. *Ex vivo* experiments incubating *S. officinale* root extract with skin microbiota from healthy human donors showed that PA derivatives underwent deacetylation and deesterification—reducing potential hepatotoxic risks without generating free alkaloids (Melnyk et al., 2023). This biotransformation, driven by bacterial enzymes, suggests topical applications may be safer than previously assumed, as the microbiota acts as a natural detoxifier. Such insights highlight the importance of integrating host-microbe dynamics into herbal dermatology research, potentially explaining interindividual variability in responses to *S. officinale*-based treatments. While these findings alleviate concerns over alkaloid-related adverse effects in external use, they also emphasize the need for microbiome-inclusive studies to optimize formulations that harness beneficial microbial transformations.

In summary, cumulative evidence positions *S. officinale* as a versatile botanical with robust skin-protective attributes, spanning antioxidant defense, wound repair, and photoprotection. Future research should explore dose-response relationships and combinatorial therapies to fully integrate *S. officinale* into evidence-based dermatological practices.

### 4.4 Anti-cancer activity

Anti-cancer activities of *S. officinale* have been evaluated using ethanolic extracts, root diets, and isolated polymers like poly[3-(3,4-dihydroxyphenyl)glyceric acid] (p-DGA), with effects observed in both whole extracts and purified compounds.

A pivotal study examined the impact of a 10% *S. officinale* extract on early hepatic carcinogenesis in Wistar rats using the resistant hepatocyte model. Chronic oral administration of the extract significantly reduced the number of macroscopic preneoplastic lesions smaller than 1 mm, diminished oval cell proliferation, and lowered mitotic activity. It also decreased proliferating cell nuclear antigen (PCNA) expression and the count of acidophilic preneoplastic nodules, while inducing cellular changes such as megalocytosis and vacuolar degeneration. Collectively, these alterations indicated suppressed cell proliferation without affecting fibrosis or glycogen storage, suggesting the extract may interfere with the initial phases of tumor promotion—potentially by modulating cellular regenerative responses (Gomes et al., 2010). This observation is striking, as it contrasts with long-term studies where *S. officinale* induces tumors, implying short-duration exposure or low doses may harness protective mechanisms against chemically induced carcinogenesis.

Building on these cellular insights, further research has explored molecular alterations induced by *S. officinale* at the gene regulatory level. Rats fed an 8% *S. officinale* root diet for 12 weeks—a regimen known to be carcinogenic—exhibited significant deregulation of microRNAs (miRNAs) and their predicted target messenger RNAs (mRNAs) in liver tissues. Notably, targeted genes were enriched in carcinogenesis-related pathways, while non-targeted genes aligned with non-cancer processes. This finding suggests *S. officinale*’s carcinogenic effects may partly stem from miRNA-mediated disruption of oncogenic networks. However, the selective targeting of cancer pathways implies potential therapeutic exploitation, provided specific miRNAs could be modulated to favor anti-tumor outcomes (Li et al., 2011). This study bridges *S. officinale*’s phytochemicals with altered gene expression via miRNA-mRNA interactions, providing a framework for understanding plant-derived compounds’ epigenetic effects in cancer contexts.

Extending beyond hepatic models, investigations into specific *S. officinale*-derived polymers have shown promising efficacy against prostate cancer. Poly[3-(3,4-dihydroxyphenyl)glyceric acid] (p-DGA) and its synthetic monomer analog (m-DGA) were tested on androgen-dependent and androgen-independent (22Rv1) human prostate cancer cells. Mechanistically, they triggered G1-phase cell cycle arrest by downregulating cyclins D1/D3/E, cyclin-dependent kinases (CDK2/4/6), and Cdc25C, alongside upregulating CDK inhibitors p21 and p27. Apoptotic induction was confirmed *via* caspase activation and PARP cleavage. Furthermore, p-DGA and m-DGA suppressed androgen receptor (AR) and prostate-specific antigen (PSA) expression, disrupting the androgen signaling critical for prostate tumor progression. *In vivo*, oral administration of p-DGA at 2.5 and 5 mg/kg body weight reduced 22Rv1 xenograft tumor volumes by 76% and 88%, respectively, in athymic mice. This was accompanied by decreased plasma PSA levels, reduced tumor PCNA and AR expression, elevated p21/p27 levels, and increased apoptosis (Shrotriya et al., 2012). These outcomes position p-DGA as a candidate for targeting hormone-refractory prostate cancer, where AR modulation and cell cycle interference could overcome resistance to conventional therapies.

Collectively, these studies illuminate *S. officinale*’s anticancer attributes through diverse mechanisms, including proliferation suppression, miRNA deregulation, and targeted apoptosis in specific cancer types. Future research should focus on purifying active fractions, elucidating structure-activity relationships, and conducting clinical trials to validate preclinical benefits, ultimately determining if *S. officinale* derivatives can be repurposed as adjunctive anticancer agents.

### 4.5 Others

Additional pharmacological properties, such as antioxidant, wound-healing, hepatoprotective, and enzyme inhibitory activities, have been demonstrated in extracts and isolated compounds from *S. officinale*, with clarifications provided on whether studies used whole extracts or purified substances.

*S. officinale* exhibits notable antioxidant activity, primarily attributed to its high content of phenolic compounds (rosmarinic acid, salvianolic acids, flavonoids). Root extracts have demonstrated robust radical scavenging capacities in DPPH, ABTS, CUPRAC, and FRAP assays, with DPPH values reaching up to 50.17 mg Trolox equivalents per gram in interspecies comparisons. Leaf extracts show even stronger DNA-protective effects against oxidative damage induced by hydrogen peroxide and ferrous ions (Lou et al., 2023; Sowa et al., 2018; Trifan et al., 2018). This antioxidant activity is enhanced by post-harvest storage, with phenolic levels peaking 1–3 months after harvest—suggesting controlled aging could amplify *S. officinale*’s utility in mitigating oxidative stress-related disorders. However, PA presence necessitates careful detoxification to maximize therapeutic benefits while eliminating toxicity risks.

Beyond antioxidant activity, *S. officinale*’s wound-healing capacity further underscores its regenerative potential—particularly in topical applications of leaf extracts. In rat models, oil-in-water emulsions containing 8% leaf extract accelerated tissue repair by increasing collagen deposition by up to 240% and reducing inflammatory infiltrates by 46% over 28 days, outperforming gel or solution formulations. Root extracts also promote fibroblast proliferation and viability in human skin cells without inducing cytoskeletal disruptions, indicating a stimulatory effect on cellular metabolism that aligns with traditional uses for fractures and ulcers (Araújo et al., 2012). This proliferative activity, combined with antioxidant support, positions *S. officinale* as a promising agent for dermal regeneration, though clinical translation requires addressing PA-related safety concerns via advanced processing techniques (Araújo et al., 2012; Sowa et al., 2018).

Notably, *S. officinale* displays hepatoprotective properties that may counteract its own potential hepatotoxicity. Certain extracts have shown superior liver-protective effects in assays, with reduced lycopsamine levels and enhanced antioxidant profiles leading to lower hepatotoxicity markers compared to conventional extracts (Syarifah et al., 2023; Trifan et al., 2018). This detoxification approach preserves bioactive phenolics while amplifying the plant’s ability to mitigate liver stress, offering insights into safer formulations for internal use. Nevertheless, long-term studies are essential to balance these benefits against residual PA risks.

**FIGURE 5:**
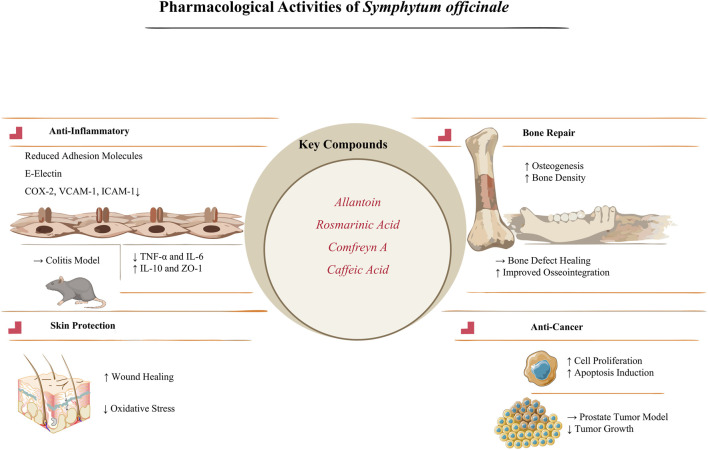
Pharmacological activities and key bioactive compounds of *S. officinale*. (Note: This schematic infographic illustrates the primary pharmacological activities of Symphytum officinale and their associated key bioactive compounds (allantoin, rosmarinic acid, comfreyn A, caffeic acid). It highlights anti-inflammatory effects (reduced adhesion molecules and modulated cytokine levels in colitis models), skin protective properties (enhanced wound healing and reduced oxidative stress), bone repair activity (promoted osteogenesis and improved osseointegration), and anti-cancer potential (inhibited cell proliferation and tumor growth in prostate cancer models), providing a visual summary of the plant’s therapeutic mechanisms).

*S. officinale*’s pharmacological profile also includes enzyme inhibitory activities with implications for managing neurodegenerative and metabolic conditions (Luca et al., 2024; Trifan et al., 2021c). Root extracts from European sources inhibit acetylcholinesterase and butyrylcholinesterase and suppress tyrosinase and glucosidase activity. Underutilized *Symphytum* species exhibit comparable or superior enzyme inhibitory effects to *S. officinale*. These activities correlate with phenolic markers (danshensu, rabdosiin) and are influenced by storage duration, with maximal potency observed shortly after harvest. Such multifaceted enzyme modulation suggests *S. officinale*’s utility in adjunct therapies for Alzheimer’s disease or diabetes, though comparative analyses highlight the value of exploring related *Symphytum* taxa to optimize bioactivity and minimize variability (Figure 5).

The bioactivities of key isolated compounds further clarify the multifaceted pharmacological profile of S. officinale. Phenolic acids, including rosmarinic acid, caffeic acid, and chlorogenic acid, exhibit strong radical-scavenging capacity in DPPH and ABTS assays and inhibit pro-inflammatory cytokine release in LPS-stimulated neutrophils (Le et al., 2021; Trifan et al., 2020). Extracts enriched with these phenolics additionally promote proliferation of human skin fibroblasts (Sowa et al., 2018). Isolated caffeic acid ethyl ester from roots inhibits E-selectin expression with an EC_50_ of 64 µM (D'Urso et al., 2020), while m-methoxybenzoic acid from root callus cultures reinforces cellular defenses against oxidative stress (Le et al., 2021). Caffeic acid oligomers such as danshensu and salvianolic acid derivatives enable synergistic anti-inflammatory interactions (Luca et al., 2024; Trifan et al., 2021b).

Lignans, particularly arylnaphthalene-type compounds such as comfreyn A, globoidnan A, globoidnan B, and rabdosiin, contribute to multiple activities. Comfreyn A inhibits E-selectin expression with an EC_50_ of 50 µM (D'Urso et al., 2020), and these lignans correlate with anti-tyrosinase and anti-glucosidase effects (Luca et al., 2024). Additional lignans, including ternifoliuslignan D, are linked to anti-apoptotic and neuroprotective properties (D'Urso et al., 2020; Le et al., 2021), while malaxinic acid enhances anti-inflammatory synergy (D'Urso et al., 2020).

Pyrrolizidine alkaloids (PAs) such as intermedine and lycopsamine show limited *in vitro* anti-inflammatory effects but are primarily associated with veno-occlusive disease *in vivo* (Luca et al., 2024). By contrast, allantoin promotes fibroblast proliferation (Sowa et al., 2018). Oxygenated fatty acids and flavonoids (e.g., quercetin-O-hexoside) support enzyme inhibition and radical quenching (Trifan et al., 2021b), and squalene augments overall antioxidant capacity (Le et al., 2021).

Polysaccharides isolated from S. officinale display antioxidant properties in radical-scavenging tests, largely attributable to uronic acid content (Shang et al., 2018). These purified fractions resist gastrointestinal digestion, are fermented by gut microbiota to produce short-chain fatty acids (SCFAs), and modulate bacterial taxa (Zhou et al., 2022). Dietary supplementation improves nutrient digestibility and laying-hen performance *via* favorable microbial shifts (Zhou et al., 2022) (Table 3).

**TABLE 3:** Summary of core pharmacological data for *S. officinale*.

Compound/Extract	Extraction type/Solvent	Dose/Concentration	Experimental model	Key quantitative results	Ref.
Comfreyn A (lignan) & Caffeic acid ethyl ester	Hydroalcoholic root (60% EtOH, PA-depleted, ethyl acetate partition)	50 µM (Comfreyn A); 64 µM (caffeic ester) (pure compounds)	IL-1β (5 ng/mL)-stimulated HUVECs	Comfreyn A: 51.5% ± 5.3% inhibition of E-selectin mRNA (EC_50_ = 50 µM); Caffeic ester: 79.6% ± 4% inhibition (EC_50_ = 64 µM)	D'Urso et al. (2020)
Root pectin (polysaccharide)	Hot-water extraction + ethanol precipitation + DEAE purification	100–400 mg/kg (extract dose, oral)	DSS-induced ulcerative colitis mouse model	Alleviated weight loss/diarrhea/colon shortening; ↓ serum TNF-α & IL-6, ↑ IL-10; ↑ ZO-1 & Muc2 expression; restored microbiota (↓ Oscillibacter/Alistipes)	Liu et al. (2024)
Leaf extract (major: rosmarinic acid 33 mg/g)	Microwave-assisted 75% methanol (750 W, 50 °C, 15 min)	125–1000 μg/mL (extract dose)	LPS (10 ng/mL)-stimulated RAW264.7 macrophages	Dose-dependent ↓ iNOS/COX-2/IL-1β/IL-6/TNF-α (protein & mRNA, ***p < 0.001 at ≥125 μg/mL); NF-κB & MAPK inactivation; DPPH IC_50_ = 110.9 μg/mL	Lou et al. (2023)
Root extract (phenolics)	Hydroalcoholic (20% EtOH w/w, mucilage-depleted)	20 μg/mL (mucilage-depleted fraction) (extract dose)	IL-1β-stimulated HUVECs	∼70% inhibition of E-selectin mRNA; blocked IKK1/2 & p65 shuttling/transactivation; ↓ COX-2	Seigner et al. (2019)
NADES leaf extract (rosmarinic acid-enriched)	Betaine-urea NADES (ultrasound-assisted, 50 °C, 45 min)	4–20 μg/mL (extract dose)	Protein denaturation (BSA/egg albumin); HepaRG hepatocytes; DPPH/ABTS	Superior ABTS IC_50_ = 0.33 μg/mL; lowest hepatotoxicity (LC_50_ 800 g/kg); strong anti-inflammatory (lowest IC_50_ vs. methanol extract)	Syarifah et al. (2023)
Root extract (for MSCs)	Not specified in study (commercial/homeopathic dilutions)	1% v/v (3C/6C/12C/30C dilutions) (extract dose)	Human bone marrow-derived MSCs (osteogenic differentiation)	↑ ALP activity (4.04-fold day 7, 7.86-fold day 21); ↑ RUNX2/osteopontin/osteocalcin; dose-dependent mineralization	Dey et al. (2020)
20% root extract cream	Aqueous/oil-in-water emulsion	10%/20% in cream (382–6123 μg/mL) (extract dose)	Rat full-thickness excisional wound model	Complete epithelialization by day 14; ↑ collagen; antimicrobial vs. *S. aureus*/*E. coli*; improved hydration/barrier	Mârza et al. (2024)
Hydroethanolic root extract	70% ethanol	2 mg/mL (extract dose)	*Ex vivo* human skin microbiota (10 donors)	PA biotransformation (deacetylation/deesterification); no free alkaloids generated	Melnyk et al. (2023)
Aqueous root extract	Hot-water	≥40 μg/mL (extract dose)	SLS-irritated human skin (*in vivo*); HaCaT/L929 fibroblasts (*in vitro*)	↓ erythema/TEWL; ↑ hydration; fibroblast proliferation without cytotoxicity	Savić et al. (2015)
Leaf extract for AgNPs	Aqueous leaf	1, 10, 100 μg/mL (extract dose)	UVB-irradiated HaCaT keratinocytes	↓ MMP-1 & IL-6; ↑ procollagen I; ROS scavenging	Singh et al. (2018)
Aqueous root extract	Aqueous	50–200 μg/mL (extract dose)	Human skin fibroblasts (MTT)	↑ proliferation & metabolism; DPPH/FRAP antioxidant; cytoskeleton preserved	Sowa et al. (2018)
p-DGA polymer (isolated)	Root (Caucasian), isolated polymer	*In vitro*: 50–100 μg/mL; *In vivo*: 2.5–5 mg/kg (pure compound)	LNCaP/22Rv1 prostate cancer cells & xenograft mice	G1 arrest (↑ p21/p27, ↓ cyclins/CDKs); ↑ apoptosis (caspase/PARP); 76%–88% tumor volume reduction	Shrotriya et al. (2012)

Collectively, *in vitro* and *in vivo* studies demonstrate plausible anti-inflammatory, osteogenic, and skin-regenerative mechanisms mediated by rosmarinic acid, allantoin, and polysaccharides. However, the majority of investigations suffer from small sample sizes, absence of blinding, lack of dose–response curves, and failure to use PA-depleted extracts—introducing confounding toxicity risks. Many cell-based assays employ supra-physiological concentrations unattainable in topical use. The data strongly support topical efficacy *via* NF-κB/MAPK inhibition and fibroblast stimulation, yet provide only weak evidence for systemic or anti-cancer effects due to inadequate pharmacokinetic data and reliance on high-dose oral models irrelevant to human topical application. Scientific quality varies widely; recent studies generally meet higher standards, while earlier work often lacks statistical rigor. While pharmacological potential is promising, critical gaps in bioavailability, long-term toxicity of refined extracts, and synergistic effects with other botanicals must be addressed through GLP-compliant, dose-optimized studies to enable evidence-based development.

## 5 Clinical applications

The clinical applications of *S. officinale* have been thoroughly assessed in randomized controlled trials (RCTs) and observational studies, with a primary focus on its topical use for musculoskeletal disorders. Prepared from *S. officinale* root extracts, topical formulations are administered externally to relieve pain, inflammation, and swelling associated with acute injuries and chronic degenerative conditions. These studies typically compare *S. officinale*-based ointments or creams to placebos or standard treatments such as diclofenac, consistently demonstrating efficacy across diverse indications alongside a favorable safety profile (Frost et al., 2013; Staiger, 2013). While this evidence supports *S. officinale*’s potential as a phytotherapeutic alternative, methodological variations and limited sample sizes in some trials warrant cautious interpretation of results (Staiger, 2012).

A prominent area of clinical inquiry centers on acute back pain, where *S. officinale* root extract has shown marked superiority over placebo. In a double-blind, multicenter RCT involving 120 patients with acute upper or lower back pain, participants applied 4 g of *S. officinale* ointment (35% root extract) three times daily for 5 days. The primary outcome, which is the area under the curve (AUC) of pain intensity during standardized movement measured *via* visual analogue scale (VAS), revealed a median reduction of 95.2% in the *S. officinale* group. This contrasts with a median reduction of just 37.8% in the placebo group (Giannetti et al., 2010). Secondary endpoints, including resting pain, pressure algometry results, and functional impairment assessed by the Oswestry Disability Index, also favored *S. officinale*, with pain relief onset observed within 1 hour (Giannetti et al., 2010). This trial’s robust design validates *S. officinale*’s potent analgesic and anti-inflammatory properties, which facilitate tissue repair. However, the short treatment duration limits insights into long-term efficacy, highlighting the need for extended follow-up studies to confirm sustained benefits.

*S. officinale*’s effectiveness also extends to knee osteoarthritis (OA), a chronic condition that impairs mobility and quality of life. In a double-blind, two-center randomized controlled trial (RCT) involving 220 patients, daily application of 6 g of *S. officinale* ointment for 3 weeks reduced the total VAS score for pain, stiffness, and functional limitation by 54.7%. This figure reflects a significant improvement compared to the 10.7% reduction observed in the placebo group (Grube et al., 2007). Findings were corroborated by the Western Ontario and McMaster Universities Osteoarthritis Index, which demonstrated enhanced mobility and higher global efficacy ratings in the *S. officinale* group (Grube et al., 2007). Similarly, multicenter trials evaluating *S. officinale* ointments blended with 10% or 20% tannic acid (administered three times daily for 6 weeks) showed both formulations outperformed eucalyptus-based placebos in reducing pain and stiffness, with no serious adverse events reported (Smith and Jacobson, 2011). These results indicate dose-dependent benefits, as higher tannic acid concentrations accelerated symptom relief (Smith and Jacobson, 2011). Critically, while these studies confirm *S. officinale*’s role in managing OA symptoms, the absence of head-to-head comparisons with oral nonsteroidal anti-inflammatory drugs in some trials leaves a gap in understanding its relative efficacy. Notably, topical administration minimizes systemic risks, a key advantage over oral alternatives.

Ankle sprains, a common acute musculoskeletal injury, represent another well-documented application for *S. officinale*, which has often matched or exceeded diclofenac in noninferiority and superiority analyses. In an observer-blind, multicenter RCT of 164 patients with unilateral ankle sprains, 2 g of *S. officinale* ointment applied four times daily for 7 days yielded a noninferior AUC for pressure pain reduction compared to diclofenac gel, with trends toward superior outcomes in swelling reduction and mobility improvement (Predel et al., 2005). A reanalysis of comparable data confirmed statistical superiority for *S. officinale*, with a mean AUC difference of 61.1 h·N/cm^2^ favoring the *S. officinale* group (D’Anchise et al., 2007). Another double-blind RCT of 142 patients reported significant pain reduction and edema reduction in the *S. officinale* group *versus* placebo over an 8 day period (Koll et al., 2004). These consistent findings across trials underscore *S. officinale*’s rapid anti-edematous effects, likely mediated by improved microcirculation and reduced inflammation (Frost et al., 2013). Nevertheless, the predominance of short-term studies and variable methodological quality call for further research into *S. officinale*’s potential for preventing recurrent sprains. This is partly due to unclear bias risks resulting from incomplete reporting in some trials.

Beyond joint-specific conditions, *S. officinale* has demonstrated utility in managing myalgia, particularly in the back. In a double-blind, multicenter RCT of 215 patients, three daily applications of a high-concentration *S. officinale* cream (10% active ingredient) achieved significantly greater pain reduction during movement and at rest compared to a lower-concentration control (Kucera et al., 2005). Faster onset of action and higher global efficacy ratings emphasize the importance of formulation strength (Kucera et al., 2005). This aligns with broader evidence supporting *S. officinale*’s versatility in treating nonspecific muscle pain, though larger patient cohorts are needed to refine subgroup analyses based on age or pain severity (Staiger, 2013).

Recent research has expanded *S. officinale*’s applications to iatrogenic complications, specifically managing enoxaparin-induced bruising in patients with acute coronary syndrome. In a double-blind RCT of 80 patients, *S. officinale* ointment reduced bruise size more effectively than placebo from days 2–5 post-intervention, with accelerated color fading indicating faster healing (Bagheri et al., 2021). This novel finding establishes *S. officinale* as a simple, safe adjunct in hospital settings, capable of improving patient comfort without interfering with anticoagulant therapy. However, the study’s focus on a specific patient population limits generalizability, and mechanistic studies are needed to clarify how *S. officinale* modulates hematoma resolution.

**FIGURE 6:**
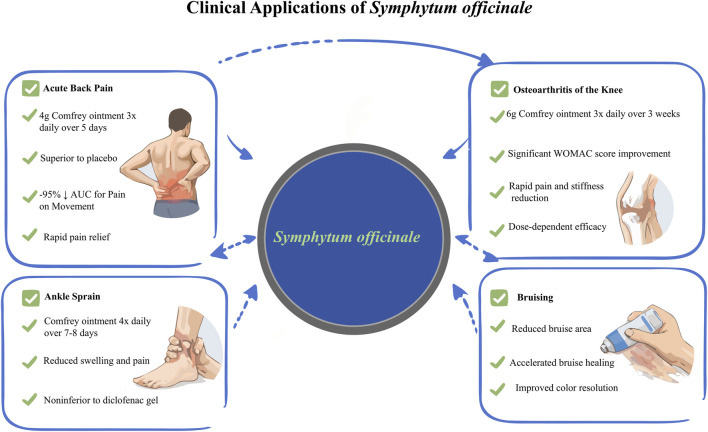
Clinical applications and evidence-based efficacy of topical *S*. *officinale* (Note: This schematic infographic summarizes the key clinical applications and evidence-based efficacy of topical *S. officinale* preparations, demonstrating their therapeutic benefits in four conditions: acute back pain (4 g ointment three times daily for 5 days, superior to placebo with rapid pain relief), ankle sprain (ointment four times daily for 7–8 days, noninferior to diclofenac gel for reducing swelling and pain), knee osteoarthritis (6 g ointment three times daily for 3 weeks, yielding significant WOMAC score improvements and dose-dependent pain reduction), and bruising (accelerated healing with reduced bruise area and improved color resolution)).

Systematic reviews synthesizing these trials confirm *S. officinale*’s efficacy for ankle sprains, back pain, wounds, and OA (Frost et al., 2013; Staiger, 2013). The high-quality RCTs in this evidence base support topical *S. officinale* as a viable alternative to synthetic anti-inflammatories, offering rapid symptom relief and improved tolerability (Oltean et al., 2014). Critically, however, the majority of RCTs are industry-sponsored (e.g., Giannetti et al., 2010; Grube et al., 2007), short-term (≤6 weeks), with high risk of performance/detection bias (Cochrane RoB assessment); no long-term follow-up or pediatric/elderly data; PA content inconsistent in older formulations. Evidence robustly confirms symptomatic relief in musculoskeletal disorders but does NOT support prevention of recurrence, efficacy in open wounds, or superiority over modern NSAIDs in head-to-head long-term trials. EMA (2015/2024) guidelines for short-term topical use on intact skin are justified, yet independent, chemically characterized RCTs are urgently needed. (Figure 6) (Table 4).

**TABLE 4:** Summary of core clinical data for *S. officinale*.

Extract type/Concentration	Dosage/Regimen	Study design	Key quantitative outcomes	References
35% root extract ointment	4 g three times daily for 5 days	Double-blind, multicenter RCT (n = 120, acute back pain)	Pain AUC (VAS movement): 95.2% reduction vs. 37.8% placebo (p < 0.001); rapid onset within 1 h	Giannetti et al. (2010)
35% root extract ointment	6 g daily for 3 weeks	Double-blind, bicenter RCT (n = 220, knee OA)	Total VAS (pain/stiffness/function): 54.7% improvement vs. 10.7% placebo; WOMAC significant improvement	Grube et al. (2007)
35% root extract ointment	2 g (6 cm strip) four times daily for 7 ± 1 day	Observer-blind multicenter RCT (n = 164, ankle sprain) vs. diclofenac gel	Noninferior/superior tenderness AUC; swelling & mobility better (mean difference 61.1 h N/cm^2^ favoring comfrey)	Predel et al. (2005), D’Anchise et al. (2007)
35% root extract ointment	2 g four times daily for 8 days	Double-blind RCT (n = 142, ankle sprain)	Significant ↓ pain & edema vs. placebo (p < 0.05)	Koll et al. (2004)
10% leaf extract cream	2–3 g three times daily for 8–10 days	Double-blind RCT (n = 215, myalgia)	Greater pain reduction (motion/rest/palpation) vs. 1% control (p < 0.05); faster onset	Kucera et al. (2005)
10% or 20% root extract + tannic acid cream	∼9.5 mg/day (3 times daily) for 6 weeks	Double-blind multiclinical RCT (n = 43, knee OA)	WOMAC pain/stiffness/function superior to placebo; dose-dependent acceleration with 20%	Smith and Jacobson (2011)
Comfrey ointment	0.3 g/cm^2^ four times daily for 5 days	Double-blind RCT (n = 80, enoxaparin-induced bruise)	Faster bruise size reduction & color resolution (days 2–5, p < 0.05) vs. placebo	Bagheri et al. (2021)

## 6 Toxicological profile

The toxicological profile of *S. officinale* has garnered significant attention due to its content of PAs—secondary metabolites linked to a range of adverse effects (Mei et al., 2010; Stickel and Seitz, 2000). Compounds such as lycopsamine, intermedine, and symphytine undergo metabolic activation in the liver, forming reactive pyrrole intermediates that bind to cellular macromolecules (e.g., DNA, proteins). This binding potential contributes to cytotoxicity, genotoxicity, and carcinogenesis (Mei et al., 2010). Experimental models have confirmed *S. officinale*’s hepatotoxic and carcinogenic properties, prompting regulatory restrictions on its internal use in many regions (Rode, 2002). For example, chronic *S. officinale* exposure in rodents induces hepatic veno-occlusive disease and tumor formation, highlighting the hazards of oral ingestion (Mei et al., 2010).

Animal studies provide compelling evidence of *S. officinale*’s toxicity. Rats fed diets containing *S. officinale* root exhibited marked changes in hepatic gene expression, with pathways involved in cellular metabolism, endothelial injury, fibrosis, and carcinogenesis being prominently dysregulated (Mei et al., 2006). Microarray analysis identified these alterations, which suggests a genotoxic mechanism for tumor initiation. *S. officinale* induced DNA adducts analogous to those produced by riddelliine, a well-characterized rodent carcinogen (Mei et al., 2006). Additionally, comparative toxicity studies in chicks exposed to PA-reduced *S. officinale* extract *versus* purified lycopsamine and intermedine showed greater toxicity with the crude extract. This was manifested as clinical signs of distress, serum biochemical abnormalities, and histopathological lesions, indicating synergistic or additive effects among PAs or their derivatives (Brown et al., 2016). Such findings underscore the potential for cumulative harm from *S. officinale*’s complex alkaloid composition, even at doses consistent with traditional use.

Translating these findings to humans, toxicity reports underscore the risks of internal *S. officinale* consumption, even though their number remains limited. Case studies have linked *S. officinale* ingestion (often *via* teas or supplements) to veno-occlusive disease, characterized by hepatic sinusoidal obstruction, ascites, and potential progression to cirrhosis (Rode, 2002; Stickel and Seitz, 2000). These incidents are associated with elevated liver enzymes and, in severe cases, fatal outcomes, though confounding factors (e.g., concurrent illnesses, co-ingestion of other hepatotoxins) complicate causal attribution. A systematic review of adverse events involving *S. officinale* and other PA-containing herbs (e.g., borage, coltsfoot) identified hepatic toxicity as the primary concern, but poor reporting quality in many cases hindered definitive conclusions (Avila et al., 2020). Notably, no comparable adverse effects have been documented with external *S. officinale* use, indicating route-dependent toxicity.

*In vitro* assays evaluating *S. officinale*’s genotoxic potential have yielded mixed results. A bacterial reverse mutation test using PA-free *S. officinale* root extract showed no mutagenic activity in *Salmonella typhimurium* strains. This was observed even with metabolic activation, indicating that purified preparations may lack the genotoxic risks associated with crude extracts (Benedek et al., 2010). However, broader metabolic analyses reveal that PAs are bioactivated by hepatic cytochrome P450 enzymes to form electrophilic species capable of inducing mutations (Mei et al., 2010). This aligns with observations of increased mutant frequencies in transgenic rat livers following *S. officinale* exposure, further supporting a genotoxic mode of action (Mei et al., 2006).

For dermal application, which is the most common modern use of *S. officinale*, pharmacokinetic investigations demonstrate minimal systemic absorption of PAs (Jedlinszki et al., 2017; Kuchta and Schmidt, 2020). In human skin models, lycopsamine penetration from *S. officinale* ointment was low: only trace amounts were detected in receptor fluids after 24 h, with even less crossing the epidermis (Jedlinszki et al., 2017; Kuchta and Schmidt, 2020). This poor bioavailability (estimated at <0.22% of the applied dose) indicates topical formulations pose substantially lower risk than oral preparations. Clinical trials and practitioner surveys corroborate this safety profile: no serious adverse events were reported in patients treated with *S. officinale* creams for back pain, ankle sprains, or OA. For instance, a multicenter trial for acute back pain found *S. officinale* ointment well-tolerated, delivering rapid pain relief without signs of systemic toxicity (Giannetti et al., 2010). Similarly, surveys of UK herbal practitioners noted frequent external use for musculoskeletal conditions, with perceived low risk for most indications—though caution was advised for open wounds or ulcers (Frost et al., 2014).

**FIGURE 7:**
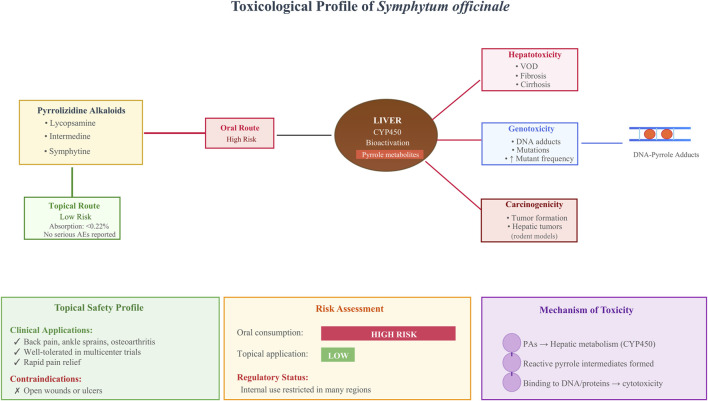
Toxicological profile of *S. officinale*: exposure routes, mechanisms of toxicity, and risk assessment. (Note: This schematic infographic outlines the toxicological profile of S. officinale, focusing on the risks associated with pyrrolizidine alkaloids. It contrasts the high risk of oral exposure with the low risk of topical application (with PA absorption of 0.04%–0.22% (Jedlinszki et al., 2017; Kuchta and Schmidt, 2020) and no serious adverse events reported), illustrates liver bioactivation via CYP450 enzymes leading to hepatotoxicity (veno-occlusive disease, fibrosis), genotoxicity (DNA adduct formation, mutations), and carcinogenicity (hepatic tumors in rodent models), and summarizes clinical safety, regulatory restrictions (internal use prohibited in many regions), and the underlying mechanism of PA-induced cytotoxicity).

Despite these reassuring findings for topical use, *S. officinale*’s overall toxicological assessment demands caution. Regulatory bodies such as the European Medicines Agency have imposed PA exposure limits, often extending oral thresholds to dermal products due to insufficient route-specific data. However, emerging absorption data challenges this equivalence, showing skin penetration is far below levels associated with hepatic damage. Non-toxicological studies also hint at potential protective mechanisms: *S. officinale* leaf extracts mitigated UV-induced damage in zebrafish models *via* ROS scavenging, which may counterbalance minor PA exposures (Cheng et al., 2014). However, long-term dermal safety data remain sparse, and inter-individual variability in skin microbiota-mediated PA metabolism is underexplored. Regulatory bodies such as the European Medicines Agency have imposed PA exposure limits, often extending oral thresholds to dermal products due to insufficient route-specific data. However, emerging absorption data challenges this equivalence, showing skin penetration is far below levels associated with hepatic damage. Older case reports (pre-2000) suffer from poor causality assessment… Route-dependent risk is clear (high oral, negligible topical with <0.22% absorption), yet cumulative low-dose effects and microbiome interactions remain unquantified. Pre-2000 animal studies (e.g., Rode, 2002) and EMA monographs (2011–2024) fully justify internal prohibition; topical safety holds when PA limits enforced (Figure 7).

## 7 Conclusion

*S. officinale* serves as a striking illustration of how traditional ethnomedicinal wisdom can align with modern scientific validation. This review provides the first comprehensive ethnopharmacological synthesis that fully integrates cross-cultural traditional knowledge with the most recent phytochemical, pharmacological, clinical, and toxicological evidence, with particular emphasis on the development and safety of PA-depleted topical formulations. For centuries, it has been valued for its anti-inflammatory, regenerative, and wound-healing properties, and contemporary research provides robust evidence to support these therapeutic effects. Phytochemical analyses have identified key bioactive components such as phenolic acids, lignans, polysaccharides, and allantoin. Clinical trials consistently demonstrate that topical *S. officinale* preparations outperform placebos or alternative treatments in relieving musculoskeletal pain, osteoarthritis symptoms, and acute injuries, with excellent tolerability profiles. Nevertheless, the presence of hepatotoxic PAs creates a critical tension between its historical benefits and modern safety concerns. This highlights the importance of restricting *S. officinale* to external use and advancing efforts to develop purified, PA-depleted extracts.

By integrating pre-2000 monographs with recent evidence and emphasizing PA-depletion, this review bridges historical wisdom and modern phytotherapy while highlighting critical safety considerations. *S. officinale* exemplifies the potential of ethnopharmacology to guide the development of safe, evidence-based phytotherapeutics. This is achievable when traditional knowledge is paired with rigorous toxicological and clinical scrutiny. By addressing gaps in prior fragmented reviews and highlighting actionable strategies for standardized, safer preparations, this work bridges traditional knowledge and evidence-based phytotherapy, offering a balanced framework for future clinical optimization.

## 8 Limitations and future research

Despite significant advances in characterizing the phytochemical composition and pharmacological activities of *Symphytum officinale*, several important limitations in the existing evidence base must be acknowledged before the plant can be fully integrated into evidence-based phytotherapy.

First, the majority of clinical trials are short-term (≤6 weeks), small-scale, and frequently industry-sponsored, limiting conclusions about long-term efficacy, safety, and prevention of recurrent musculoskeletal conditions. Most randomized controlled trials focus exclusively on topical applications for acute back pain, knee osteoarthritis, and ankle sprains, leaving potential benefits for other traditional indications (e.g., gastrointestinal or respiratory disorders) largely untested in humans. Second, phytochemical and pharmacological studies often rely on crude or inconsistently standardized extracts, with poor control of PA content and batch-to-batch variability; this reduces reproducibility and complicates regulatory acceptance. Structure–activity relationships for many minor constituents remain tentative, and few investigations employ GLP-compliant methods or metabolomic fingerprinting across geographic origins. Third, toxicological data are predominantly derived from high-dose oral animal models or historical case reports with confounding factors, while long-term dermal safety, cumulative low-dose effects, and inter-individual variability in skin-microbiota-mediated PA metabolism are under-explored. Finally, ethnopharmacological surveys suffer from recall bias, lack of standardised preparation methods, and underrepresentation of non-European traditions, while preclinical mechanistic studies frequently use supra-physiological concentrations that may not reflect realistic topical exposure.

These limitations notwithstanding, the current body of evidence still offers a solid foundation for advancement. To address these gaps, future research should prioritize standardized protocols to enhance comparability and reliability. This includes comprehensive metabolomic profiling to account for geographical and seasonal fluctuations in plant composition, which can inform the refinement of PA-depletion techniques. Sustainable methods—such as selective breeding or enzymatic treatments—should be explored to develop safer *S. officinale* variants without compromising efficacy (Przybylska et al., 2022; Zhou et al., 2013). Parallel to this, expanding clinical research through multi-center, long-term randomized controlled trials are essential. These trials should include diverse patient cohorts to evaluate broader therapeutic indications and optimal dosing, while comparing *S. officinale* derivatives to conventional treatments to establish relative advantages (Nazarova et al., 2022; Susilawati et al., 2023). Synergistic formulations—blending *S. officinale* with complementary botanicals—could also be investigated to amplify effects in wound healing or anti-cancer contexts, addressing the current overemphasis on isolated extracts (Neagu et al., 2023). Additionally, interdisciplinary collaborations that integrate ethnopharmacological heritage with advanced technologies hold promise for unlocking novel applications. These range from prebiotic supplements to advanced dermatological therapies, with technologies such as CRISPR for genetic optimization or nanotechnology for targeted drug delivery (Glaser et al., 2017; Yan et al., 2025).

The reviewed data convincingly validate traditional topical uses for musculoskeletal complaints and wound healing, yet reveal critical limitations: predominance of descriptive ethnobotany, variable phytochemical standardization, short-duration clinical trials, and insufficient long-term safety/toxicokinetic studies. Technological relevance as a sustainable crop and cosmetic ingredient is underexploited due to regulatory hurdles. Future research must prioritize multi-center, independent RCTs with chemically defined extracts, metabolomic-guided standardization, microbiome-PAs interaction studies, and development of CRISPR-edited low-PA varieties. Only through such rigorous, critical approaches can *S. officinale* fully transition from traditional remedy to safe, evidence-based phytotherapeutic and industrial resource.
